# Sensitive and resistant of the homologous disulfide-bridged proteins α-lactalbumin and lysozyme to attack of hydrogen-atoms, dithiothreitol and trifluoroacetic acid, examined by matrix-assisted laser desorption/ionization mass spectrometry

**DOI:** 10.1016/j.bbrep.2022.101212

**Published:** 2022-01-24

**Authors:** Mitsuo Takayama

**Affiliations:** Graduate School of Nanobioscience, Yokohama City University, 22-2 Seto, Kanazawa-Ku, Yokohama, 236-0027, Japan

**Keywords:** Bovine α-lactalbumin, Hen egg-white lysozyme, Backbone cleavage, Disulfide-bridge, Acid hydrolysis, MALDI mass spectrometry

## Abstract

**Background:**

Evolutionarily homologous proteins bovine α-lactoalbumin (αLA) and hen egg-white lysozyme (HEL) are very similar in primary, secondary and tertiary structures involving the location of disulfide-bridges (S–S), and are resistant to the action of hydrolytic enzymes and reagents. It is of interest to examine and compare the difference in backbone cleavage characteristics, by using reductive and hydrolytic reagents.

**Methods:**

In-source decay (ISD) combined with matrix-assisted laser desorption/ionization mass spectrometry (MALDI MS), reductive treatment of αLA and HEL with dithiothreitol (DTT) and acid hydrolysis with trifluoroacetic acid (TFA) were employed to examine the difference in the backbone cleavage characteristics of αLA and HEL.

**Results:**

The treatment of αLA and HEL with DTT/AcOHNH_3_ resulted in similar cleavage behaviors of the backbone N-Cα and S–S bonds, i.e., the enhancements of the intensity and *m/z* range of sequence-reflected fragment ions were very similar. However, the treatment of αLA with DTT/TFA resulted in unexpected residue-specific degradation at the peptide bond of the Asp-Xxx, Xxx-Ser/Thr, Gln-Xxx, Xxx-Gly and Gly-Xxx residues, while HEL did not occur such degradation.

**Conclusions:**

The results obtained above indicate that acidic αLA is very sensitive to acidic additive such as TFA, while basic HEL is resistance to acidic additives.

**General significance:**

The study demonstrates the sensitive and resistant of evolutionary homologous proteins αLA and HEL to the acid hydrolysis and these characters come from acidic and basic nature of the proteins.

## Highlights

• Evolutionary homologous proteins bovine alpha-lactoalbumin and hen egg-white lysozyme are quite different in the protection from acidic reagents.
• Alpha-lactoalbumin is easily hydrolyzed with acidic reagents at the specific Asp, Gly, Thr and Ser residues owing to the acidic protein with pI4.53
• Lysozyme is perfectly resistant to acidic reagents due to the presence of strong basic Arg residues owing to the basic protein with pI10.7
• Degradation characteristics at the backbone S–S and N-Cα bonds of both proteins are very similar.

## Graphical abstract

**Figure:**
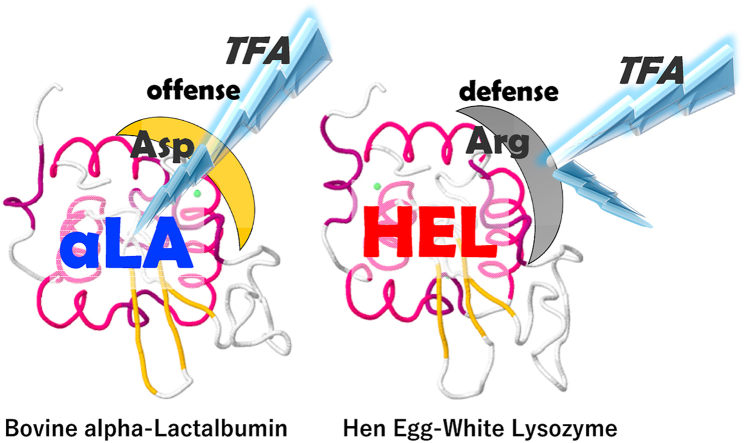

## 1 Introduction

**Fig. 1:**
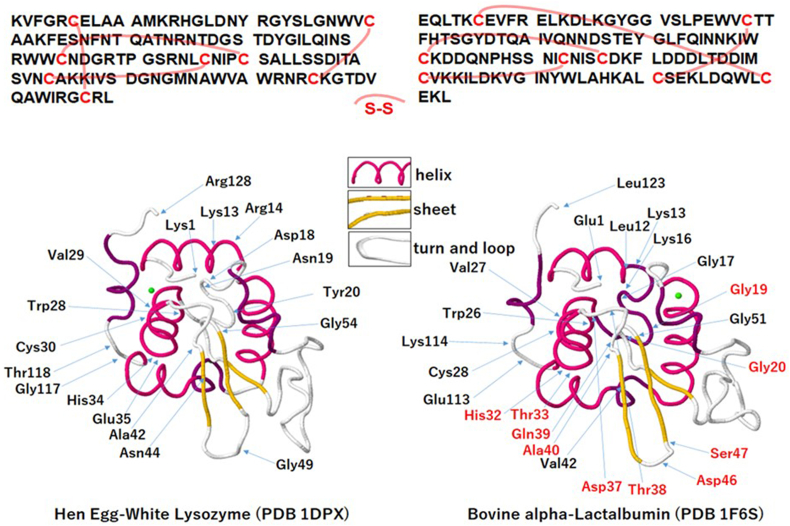
Structural information of hen egg-white lysozyme and bovine alpha-lactalbumin. Upper, primary and disulfide bridge (S–S), lower, the secondary and tertiary structures (the inset represents color legend for secondary structures, helix, sheet, turn and loop). (For interpretation of the references to color in this figure legend, the reader is referred to the Web version of this article.)

Of the many possible post-translational modifications of proteins, disulfide-bridges (S–S) play an important role for stabilizing higher order structures and specific biological activity [[1], [2], [3]], and the S–S bonds present difficulties in analysis with bond cleavages due to its stability [4]. Comparative study of the evolutionary homologous proteins, bovine α-lactoalbumin (αLA) and hen egg-white lysozyme (HEL) containing four S–S bonds, is of longstanding interest in the biological sciences [5], and has been performed from the standpoint of conformational studies based on X-ray crystallography [6], folding intermediates based on CD [7], unfolded structures based on NMR [8] and oxidative folding [9]. The properties of these proteins have been compared and summarized in the review article by the Brew group [5]. According to the reports, bovine αLA and HEL are very similar in secondary and tertiary structures including sites of S–S bonds (Fig. 1), whereas function, namely enzymatic hydrolysis of HEL and Ca^2+^ ion binding affinity of bovine αLA are different. It is also a common characteristic that both αLA and HEL are highly resistant to digestive hydrolysis. It is noteworthy, on the other hand, that iso-electric point (pI) of αLA is quite different from that of HEL, coming from the number of acidic Asp and basic Arg residues (Table 1).

**Table 1:** Information of hen egg-white lysozyme and bovine alpha-lactalbumin, relative-molecular mass (*M*r), iso-electric point (pI), number (Nr) of basic and acidic amino acids, and sites of S–S bond.

Hen egg-white lysozyme		Bovine α-Lactalbumin
*M*r	14305.1	14178.0
pI	10.7	4.53
Nr of Arg	11	1
Nr of Lys	6	12
Nr of His	1	3
Nr of Glu	2	7
Nr of Asp	7	13
Sites of S–S	6-127, 30–115, 64–80, 76-94	6-120, 28–111, 61–77, 73-91

It should be noted that researchers have interested in the hydrolytic products of αLA owing to the bioactive functions [10,11], whereas HEL is not target of interest in the hydrolysis. This may be due to sensitive and resistant nature of αLA and HEL to the action of protease and reagent for hydrolysis. Therefore, it is of importance to examine and compare the degradation characteristics of αLA and HEL. I recently reported that in-source decay (ISD) coupled with matrix-assisted laser desorption/ionization mass spectrometry (MALDI MS) results in discontinuous intense fragment ions originating from cleavage of the N-Cα bond of the Xxx-Asp/Asn, Xxx-Cys and Gly-Xxx residues of disulfide-bridged and phosphorylated proteins [12]. The MALDI-ISD is a method for specifically cleaving the N-Cα bond of the backbone of intact proteins, by attacking the backbone carbonyl oxygen with hydrogen atoms generated from excited matrix molecules [13,14]. It is of importance to recognize that MALDI-ISD can reduce the disulfide-bridge (S–S) to form sulfhydryl groups (-SH HS-) and simultaneously cleave at the N-Cα bond of disulfide-bridged proteins [12]. It is of interest to compare the cleavage characteristics of evolutionarily homologous proteins αLA and HEL, because it may be expected that αLA would have different cleavage properties from the highly evolutionarily conserved enzyme HEL which is resistant to the action of reductive and acidic reagents, and it would give useful information for relationship between cleavage susceptibility and physicochemical nature such as basic and acidic properties, and for the study of amyloid fibril formation of αLA and HEL [[15], [16], [17]].

This paper examines the cleavage characteristics of αLA and HEL using MALDI MS, by treating the proteins with the reagents such as dithiothreitol (DTT), trifluoroacetic acid (TFA) and acetic acid (AcOH). Here I found that treatment of αLA by DTT with TFA resulted in unexpected residue specific hydrolytic degradation, while HEL did not occur such degradation. The influence of DTT on the simultaneous cleavages of the N-Cα and S–S bonds of both proteins with ISD experiments is also examined from the standpoints of enhancement of the intensity and *m/z* range of sequence reflected fragment ions.

## 2 Materials and methods

### 2.1 Reagents

5-Amino-1-naphthol (5,1-ANL), dithiothreitol (DTT), acetic acid (AcOH) and trifluoroacetic acid (TFA) were purchased from Tokyo Chemical Industry (Tokyo, Japan). Acetonitrile and ammonium acetate (AcOHNH_3_) were purchased from Fujifilm Wako Pure Chemicals (Tokyo, Japan). Water used in all experiments was purified using a MilliQ water purification system from Millipore (Billerica, MA, USA). Bovine α-lactoalbumin (αLA) and hen egg-white lysozyme (HEL) were purchased from Sigma-Aldrich (Steinheim, Germany). All reagents were used without further purification. Each protein for MALDI MS experiments was dissolved 200 μL of water at a concentration of 100 μM in a 600 μL microtube. 5 mg of each matrix was dissolved in 200 μL of a solvent of water/acetonitrile (3:7, v/v). The matrix and analyte solutions were prepared without any additives such as trifluoroacetic acid. Sample solution was prepared by mixing 10 μL of analyte solution with 10 μL of matrix solution using a shaker just before MALDI-ISD experiments were performed. A volume of 1.5 μL of the sample solution was deposited onto a stainless-steel MALDI target plate using a 10 μL micropipette and the solvents removed by allowing evaporation in air at room temperature. To reductively cleave the disulfide-bridges of analytes, a solution of DTT was prepared at 500 mM in water mixed with ammonium acetate (AcOHNH_3_) as a buffer, and 10 μL of DTT solution was mixed with 200 μL of analyte solution in water. The prepared sample solution was incubated for 2.5 h at 25 °C.

### 2.2 Mass spectrometry

MALDI mass spectra were acquired on a time-of-flight mass spectrometer AXIMA-CFR (Shimadzu, Kyoto, Japan) equipped with a nitrogen laser (337 nm wavelength) operating at a pulse rate of 10 Hz. The pulse width of the laser was 4 ns. The laser spot size on the target substrate was ca. 100 μm in diameter. The ions generated by MALDI were accelerated using 20 kV with delayed extraction. The analyzer was operated in the linear and reflector mode and the ions were detected using a secondary electron multiplier. A total of 500 shots were accumulated for each mass spectrum acquisition.

## 3 Results and discussion

### 3.1 Simultaneous S–S and N-Cα bond cleavage of αLA and HEL with and without DTT

**Fig. 2:**
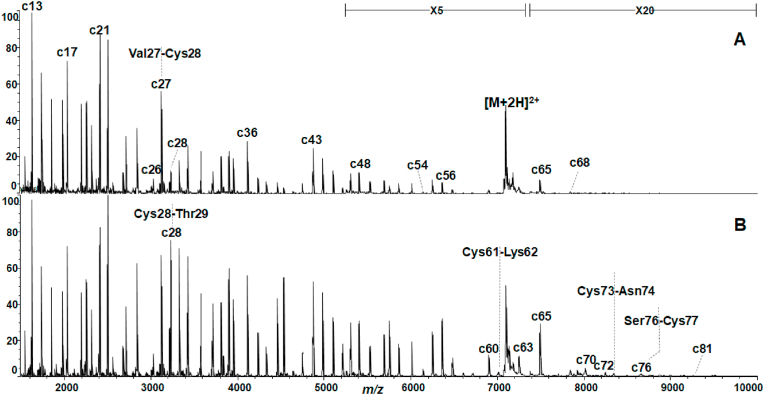
MALDI mass spectra of bovine αLA obtained (A) without and (B) with DTT/AcOHNH_3_ at 25 °C for 2.5 h of incubation. The vertical axis represents relative intensity (%) of c ions in the *m/z* range indicated.

**Fig. 3:**
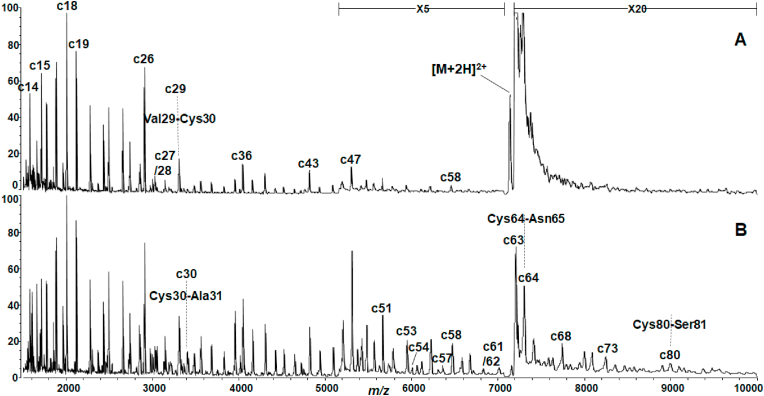
MALDI mass spectra of HEL obtained (A) without and (B) with DTT/AcOHNH_3_ at 25 °C for 2.5 h of incubation. The vertical axis represents relative intensity (%) of c ions in the *m/z* range indicated.

MALDI mass spectra of bovine αLA and HEL with/without DTT are shown in Fig. 2, Fig. 3, respectively. The MALDI mass spectra of both proteins showed a lot of fragment ions called as c-series ions originating from cleavage of the backbone N-Cα bonds. The formation of the c-series and related fragment ions is illustrated in Fig. S1. The nomenclature of the ISD fragment ions obey the recommendation by Chu et al. [18], and the formation mechanism of the fragment ions has been reported elsewhere [[12], [13], [14]]. As already reported [12], it is known that MALDI-ISD results in specific sensitive cleavage at the backbone N-Cα bond of Xxx-Cys, Xxx-Asp/Asn, Xxx-Ser and Gly-Xxx residues, while Xxx-Ile/Val residues are insensitive to cleavage. The spectrum of αLA showed discontinuous intense peaks of c13, c17, c21, c27, c36 and c43 ions originating from cleavage at the N-Cα bond of Lys-Asp, Gly-Tyr, Val-Ser, Val-Cys, Tyr-Asp and Gln-Asn residues, respectively. With HEL, intense peaks of c18, c26, c29, c36 and c43 ions were observed originating from cleavage of Asp-Asn, Gly-Asn, Val-Cys, Ser-Asn and Thr-Asn residues, respectively. The MALDI mass spectra of both proteins clearly showed c43 ions originating from cleavage of Xxx-Asn44 (for αLA) and Xxx-Asn44 (for HEL). This indicates that MALDI-ISD results in reductive cleavage of the disulfide-bridges such as Cys6-Cys120 and Cys28-Cys111 in αLA and Cys6-Cys127 and Cys30-Cys115 in HEL, as well as cleavage of the backbone N-Cα bonds. It can be presumed that the probability of simultaneous cleavage of both S–S and N-Cα bonds is lower than for cleavage of the N-Cα bond alone. In fact, a drop in the peak intensity of the c28 ion from bovine αLA (Fig. 2A) and the c30 ion from HEL (Fig. 3A) was observed for the bridged sites of Cys28-Cys111 and Cys30-Cys115, respectively, when DTT was not used.

As shown in Fig. 2B, reductive treatment of αLA with DTT resulted in the enhancement of the peak intensity and *m/z* range from c28 to c81 ions. The enhancement of the intensity of the c28 ion clearly indicates that the treatment with DTT results in cleavage of the S–S bond between Cys28 and Cys111. Similarly, the enhancement of the intensity and *m/z* range can be confirmed from observation of the c ions from c30 to c80 in the spectrum of HEL (Fig. 3B). The influence of DTT treatment on the peak intensity of c ions can be quantitatively estimated from the ratio of the intensity of c ions. The c-series ions observed in the spectra of αLA and HEL increased upon treatment with DTT at c28 and c30, respectively, while c ions in samples lacking DTT discontinuously dropped in peak intensity at c28 and c30. For αLA in the absence of DTT, the ratio of the sum-total of intensity of c28 to c37 to that of c17 to 27 was 0.46, while with DTT the ratio was 0.90 (Fig. S2). Similarly, the ratios for HEL without and with DTT were 0.29 and 0.63 (Fig. S3), respectively. The ratios obtained indicate that for both proteins the intensity of c ions is enhanced approximately 2 folds by treatment with DTT. Furthermore, the treatment with DTT resulted in the enhancement of the *m/z* range of observed c ions, as shown in Fig. 2, Fig. 3. With bovine αLA, the c60 to c81 peak ions are clearly observed upon reductive cleavage of S–S bonds (Fig. 2B), and in HEL the c58 to c80 peak ions are clearly observed (Fig. 3B).

Furthermore, the MALDI mass spectra of bovine αLA and HEL gave informative fragments, namely C-terminal y, z and w ions (Fig. S1) which can be used for elucidating the sites of cleavage of the S–S bonds. In particular, the observation of w ions gives strong evidence for the presence and cleavage of the S–S bond. The ISD fragment z, w and c ions observed in the MALDI mass spectra of both proteins obtained with/without DTT are summarized in Table 2. For αLA, the observation of z10 to z40 ions and w13 and w33 ions indicates that Cys6-Cys120, Cys28-Cys111 and Cys73-Cys91 were cleaved by ISD with/without DTT. For HEL, with DTT the observation of z7 to z60 ions and w15, w36 and w54 ions gives evidence for cleavage at the S–S bond of all Cys-Cys residues (Cys6-Cys127, Cys30-Cys115, Cys64-Cys80, Cys76-Cys94). As shown in Table 2, the observation of c7 to c68 ions with αLA and c6 to c58 ions with HEL in the absence of DTT indicates that S–S bonds at Cys6-Cys120, Cys28-Cys110 and Cys61-Cys73 of α-LA and Cys6-Cys127 and Cys30-Cys115 of HEL are cleaved. Although this suggests that disulfide-bridges in HEL molecules may be more resistance to attack by matrix hydrogens than those of αLA molecules, especially in at Cys64-Cys80 of HEL, the treatment with DTT resulted in very similar behavior with respect to the enhancement of the intensity and *m/z* range of c ions.

**Table 2:** ISD fragment z, w and c ions observed in MALDI mass spectra of αLA and HEL obtained with and without (wo) DTT.

Fragment	αLA	HEL
Z	z10 to z40 (wo DTT)	z7 to z29 (wo DTT)
	z10 to z40 (with DTT)	z7 to z60 (with DTT)
W	w13, w33 (wo DTT)	w15, w36 (wo DTT)
	w13, w33 (with DTT)	w15, w36, w54 (with DTT)
C	c7 to c68 (wo DTT)	c6 to c58 (wo DTT)
	c7 to c81 (with DTT)	c6 to c81 (with DTT)

### 3.2 Unexpected residue-specific degradation of αLA with DTT

**Fig. 4:**
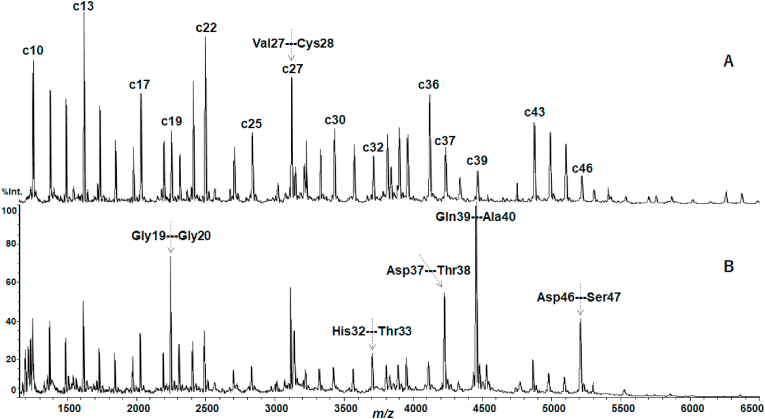
MALDI mass spectra of αLA obtained with DTT/AcOHNH_3_ at 25 °C for (A) 10 min and (B) 4 days of incubation. The vertical axis represents relative intensity (%) of c ions in the *m/z* range indicated.

As described above, the backbone cleavage characteristics of αLA prepared with DTT were very similar to those of HEL. Here I found, however, that standing the prepared solutions for a couple of day at room temperature resulted in unexpected degradation of αLA (Fig. 4B), while HEL did not occur such degradation even when TFA was used as is described later. Fig. 4 shows the comparison of MALDI mass spectra obtained by incubation for 10 min and 4 days under the conditions with DTT/AcOHNH_3_ at 25 °C. Four days of incubation resulted in the appearance of some intense peaks (Fig. 4B), whereas the spectrum obtained by incubation for 10 min showed a usual ISD pattern (Fig. 4A). The observed intense peaks originating from degradation of αLA were overlapped with ISD peaks of c19, c32, c37, c39 and c46 ions corresponding to cleavage at the N-Cα bond of the Gly19-Gly20, His32-Thr33, Asp37-Thr38, Gln39-Ala40 and Asp46-Ser47 residues, respectively (Fig. 4). From the cleavage residues such as Gly, Thr, Ser and Asp, it is suggested that acid hydrolysis takes place at the backbone amide (C–N) bond [19,20], although any acidic additives were not used in this experiment except for ammonium acetate as a buffer.

**Fig. 5:**
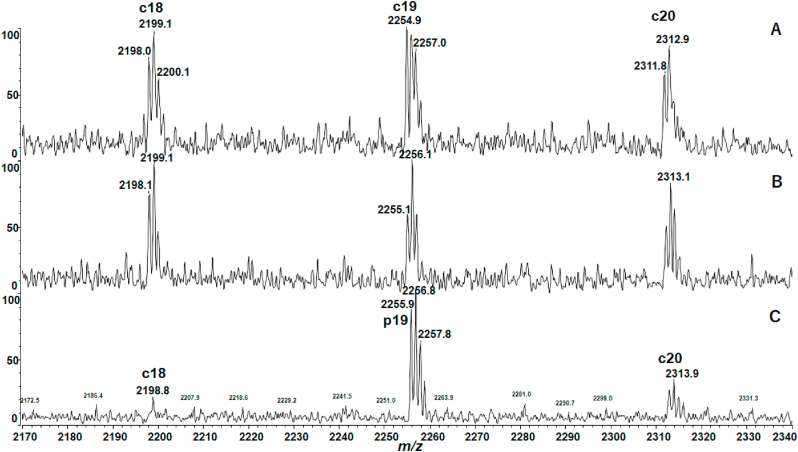
Partial MALDI mass spectra of c18 (*M*m 2198), c19 (*M*m 2255) and c20 (*M*m 2312) ions of αLA obtained with DTT, (A) without added TFA, and with added TFA for (B) 30 min and (C) 24 h at 25 °C. The vertical axis represents relative intensity (%) of c ions in the *m/z* range indicated.

**Fig. 6:**
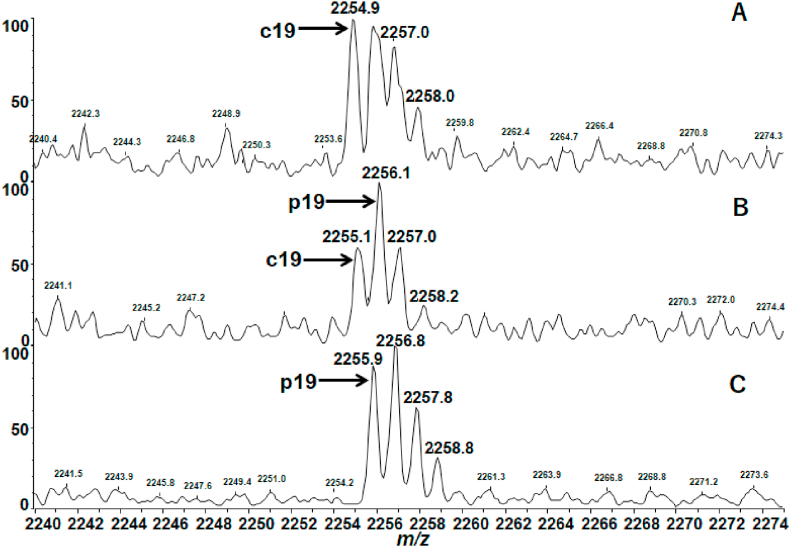
Enlarged MALDI mass spectra of c19 (*M*m 2255) and p19 (*M*m 2256) ions generated from the backbone cleavage of αLA obtained with DTT, (A) without added TFA, and with added TFA for (B) 30 min and (C) 24 h at 25 °C. The vertical axis represents relative intensity (%) of c ions in the *m/z* range indicated.

To examine the possibility of hydrolytic degradation with acids, the MALDI MS experiments were performed after preparation with added TFA (0.5% v/v) to αLA and HEL solutions with DTT. The MALDI mass spectra of αLA showed extremely intense degraded peaks overlapped with c19, c32, c37, c39 and c46 ions (Fig. S4). On the other hand, the MALDI mass spectrum of HEL obtained by incubation for 4 h with DTT/TFA at 25 °C merely showed c ions originating from cleavage at the N-Cα bond which characterize the ISD reaction differing from the acid hydrolysis (Fig. S5). From the MALDI mass spectra in Fig. S4, it should be noted here that a hydrolytic product ion p_n_ with the C-terminal structure (-COOH, 45 Da) could not be separated from the corresponding n-th c_n_ ion with the structure (-C(OH)NH, 44 Da) owing to the liner mode experiments. To separate and identify the p_n_ ion from c_n_ ion, the MALDI MS experiment with high resolution reflector mode was performed by selecting c19 ion (*m/z* 2255 in monoisotopic mass) and the adjacent c ions (Fig. 5). Fig. 5B and C obtained with DTT/TFA for 30 min and 24 h, respectively, showed an intense peak at *m/z* 2256 corresponding to monoisotopic mass (*M*m) of the hydrolytic product p19, while Fig. 5A obtained without TFA showed isotope pattern of c19 ion. The change in isotope peak pattern from c19 to p19 in Fig. 6 indicates that the treatment of αLA with TFA resulted in acid hydrolysis of the C–N bond of the Gly19-Gly20 residues. The results obtained indicate that αLA is sensitive to TFA and easily takes place residue-specific degradation of the Gly-Gly, His-Thr, Asp-Thr, Gln-Ala and Asp-Ser residues, while HEL is resistant to TFA. It was also confirmed that the treatment of αLA with acetic acid resulted in same degradation (data not shown).

The result obtained above strongly suggests that unexpected degradation of αLA is the acid hydrolysis which is characterized by residue-specific cleavage at the peptide bond of the Asp-Xxx, Xxx-Ser/Thr, Xxx-Gly and Gly-Xxx residues [16,17], though the hydrolysis of the Gln-Ala residues has not been reported so far. Interestingly, Rahamtullah and Mishra recently reported that in the study of amyloid fibril formation from αLA the acidic condition at 65 °C resulted in hydrolytic nicking and fragmentation of the peptide bond of Asp-Xxx residues [15], although the fragment ions they reported were not in accord with the intense fragment ions observed in Fig. 4B. This inconsistency of fragment ions may be due to the experimental conditions such as acidic additives (HCl and TFA) and solution temperature (25 °C and 65 °C), especially elevated temperature 65 °C Rahamtullah and Mishra employed may be accelerating bond cleavage reactions. Regarding this, further experimental study is needed.

**Fig. 7:**
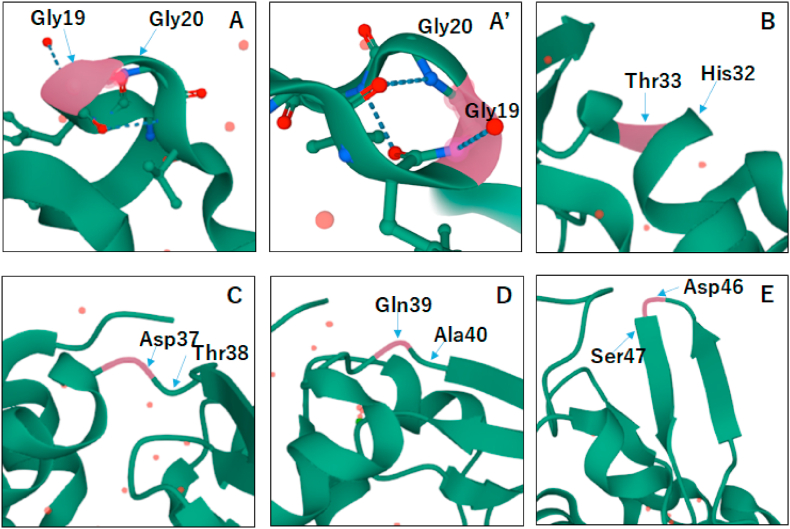
The sites of the acid hydrolytic degradation residues in bovine αLA (PDB: 1F6S).

Regarding the mechanistic considerations of unexpected degradation here, from the secondary structure of the sites of hydrolysis such as Gly19-Gly20, His32-Thr33, Asp37-Thr38, Gln39-Ala40 and Asp46-Ser47 residues of αLA (Fig. 7), it is likely that the sites are easily attacked by acidic TFA molecules because the backbone amide of the sites expose from the surface of αLA molecules. It is also interesting that the degraded sites described above are lying in turn and loop regions in secondary structure (see the residues indicated by the red color in Fig. 1), as is known from the atomic resolution structure of αLA [21]. With respect to the observation of degraded intense peaks in Fig. 4B, it may be presumed that hydrolysis occurs by acetic acid AcOH separated from the buffer AcOHNH_3_ added with DTT, while counter ammonia molecules NH_3_ may be trapped by the acidic residues (Asp and Glu) exposed from the surface of αLA which is an acidic protein with pI4.53. In other words, αLA molecules cannot be protected from the attack of TFA or other acidic reagents, because αLA has little strong basic Arg residues for forming salt-bridge with TFA. The reason why HEL does not occur the acid hydrolysis is that HEL can trap TFA or form salt-bridge with TFA through the basic Arg and Lys sidechains exposed from the surface of HEL which is a basic protein with pI10.7. It should be noted here that HEL also undergoes hydrolytic degradation when acidic, elevated temperature and long-time incubation were employed, as reported by Frare et al. [16], although the fragments they reported were not observed in the MALDI mass spectra of HEL obtained with DTT/AcOHNH_3_ and DTT/TFA at 25 °C (see Fig. S5). This inconsistency also may be due to the experimental conditions such as acidic additives, solution temperature and incubation time, as described above. As a result, it should be stressed that αLA is quite different from HEL in the sensitivity to attack of acids, owing to the acidic and basic nature of αLA and HEL, respectively.

### 3.3 Cleavage characteristics based on the intensity ratio of c ions of αLA and HEL

The intensity ratio of c ions in MALDI-ISD spectra of peptides and proteins gives a measure of the extent of residue specific cleavage at the N-Cα bond [12]. The intensity ratio R (Cn) for n-th c ion can be defined by the ratio of the intensity Int (Cn) of n-th c ion to the average intensity of the adjacent side c-ion peaks as follows.

(1) R (Cn) = Int (Cn)/(Int (C_n-1_) + Int (C_n+1_))/2

**Fig. 8:**
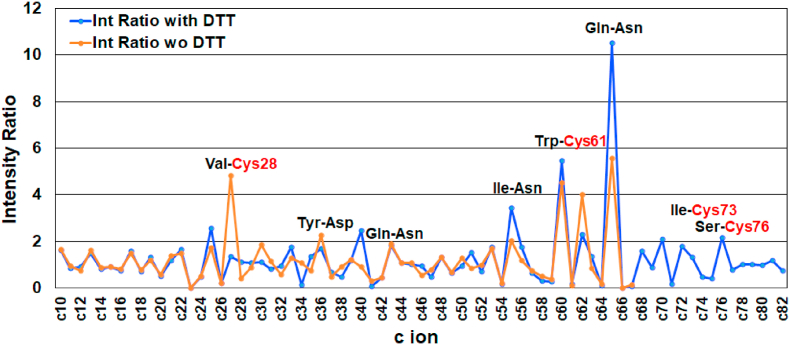
Intensity ratio of c ions obtained from MALDI-ISD of bovine αLA with/without DTT.

**Fig. 9:**
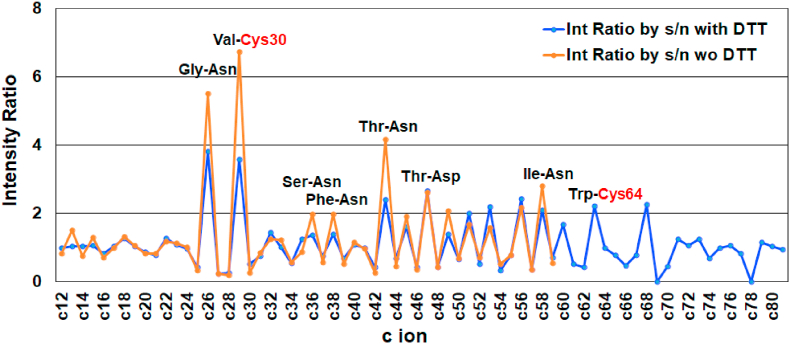
Intensity ratio of c ions obtained from MALDI-ISD of HEL with/without DTT.

The intensity ratios of c ions calculated from the spectra in Fig. 2, Fig. 3 for αLA and HEL with/without DTT are shown in Fig. 8, Fig. 9, respectively. For both proteins, the intensity ratios corresponding to cleavage at the N-Cα bond of the Xxx-Cys and Xxx-Asp/Asn residues are extremely high independent of treatment with DTT, while the intensity ratio for the Xxx-Pro residue is 0 owing to incomplete cleavage. For αLA, the total average of the intensity ratio with and without was 1.07 and 1.08, respectively, while in HEL the average with and without was 1.04 and 1.09, respectively. The extremely high values of R (Cn) > 4.0 for Xxx-Cys and Xxx-Asn and R (Cn) = 0 for Xxx-Pro were removed from calculations to avoid unnatural deviation. The results obtained here indicate that treatment with DTT does not influence the cleavage characteristics of the N-Cα bond for either protein. Furthermore, the residue specific cleavage character of αLA is almost the same in terms of intensity ratio as HEL.

## 4 Conclusions

The cleavage characteristics of the backbone S–S, N-Cα and C–N bonds of homologous disulfide-bridged proteins bovine α-lactoalbumin (αLA) and hen egg-white lysozyme (HEL) have been examined by MALDI-ISD method, with added the reagents DTT, AcOHNH_3_ and TFA. Although the treatment of αLA and HEL with DTT/AcOHNH_3_ resulted in similar enhancement of the intensity and *m/z* range of c ions in both proteins, the acidic protein αLA occurred unexpected degradation at the Gly-Gly, His-Thr, Asp-Thr, Gln-Ala and Asp-Ser residues, whereas HEL did not occur such degradation. The treatment of αLA with DTT/TFA suggested that the acid hydrolysis of the specific residues described above. It might be assumed that the sensitivity of αLA and resistance of HEL to TFA are due to the acidic nature of αLA and basic nature of HEL, respectively. Although the clear-cut evidence for this argument could not be obtained here, it would be verified by shifting the pI of both HEL and αLA using site-directed mutagenesis and modifications.

## Credit author statement

M.T. conceptualized the project, performed the MALDI MS experiments, analyzed the mass spectra and wrote the manuscript.

## Declaration of competing interest

The author declares no competing financial interest.

## Data Availability

No data was used for the research described in the article.
