# Supplementary material for: Sensitive and resistant of the homologous disulfide-bridged proteins α-lactalbumin and lysozyme to attack of hydrogen-atoms, dithiothreitol and trifluoroacetic acid, examined by matrix-assisted laser desorption/ionization mass spectrometry
Source: Biochem Biophys Rep. 2022 Jan 24;29:101212. doi: 10.1016/j.bbrep.2022.101212 (PMC8790284; doi:10.1016/j.bbrep.2022.101212)
Supplement: Multimedia component 1 [file mmc1.docx]

**Supplementary data**

Sensitive and resistant of the homologous disulfide-bridged proteins a-lactalbumin and lysozyme to attack of hydrogen-atoms, dithiothreitol and trifluoroacetic acid, examined by matrix-assisted laser desorption/ionization mass spectrometry

Mitsuo Takayama

Graduate School of Nanobioscience, Yokohama City University, 22-2 Seto, Kanazawa-Ku, Yokohama 236-0027, Japan.

E-Mail: [takayama@yokohama-cu.ac.jp](mailto:takayama@yokohama-cu.ac.jp)

**Fig. S1**. Nomenclature and formation mechanism of ISD fragment ions. In text the notation of c, z, y and w ions is used for simplicity.


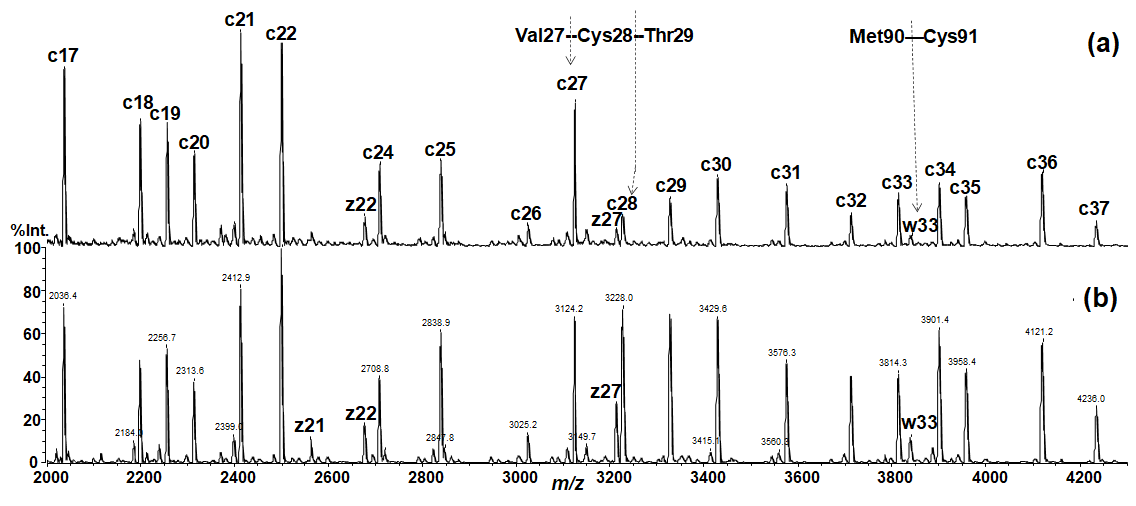


**Fig. S2**. MALDI mass spectra of bovine αLA obtained by 5,1-ANL matrix (a) without and (b) with DTT, used for calculating the ratio of the sum-total of intensity of c28-c37 to that of c17-c27.


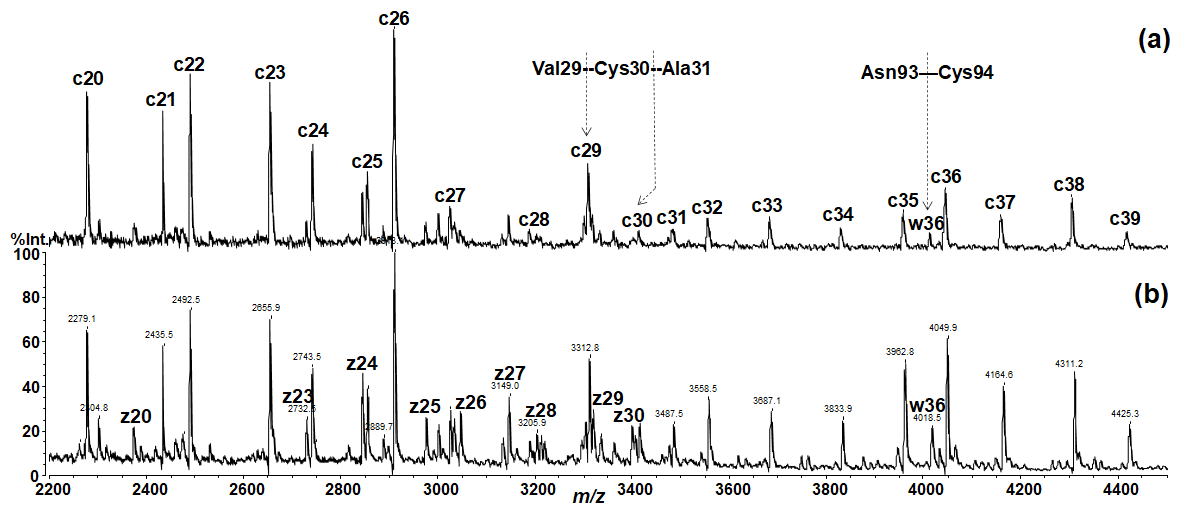


**Fig. S3**. MALDI mass spectra of HEL obtained by 5,1-ANL matrix (a) without and (b) with DTT, used for calculating the ratio of the sum-total of intensity of c30-c39 to that of c20-c29.


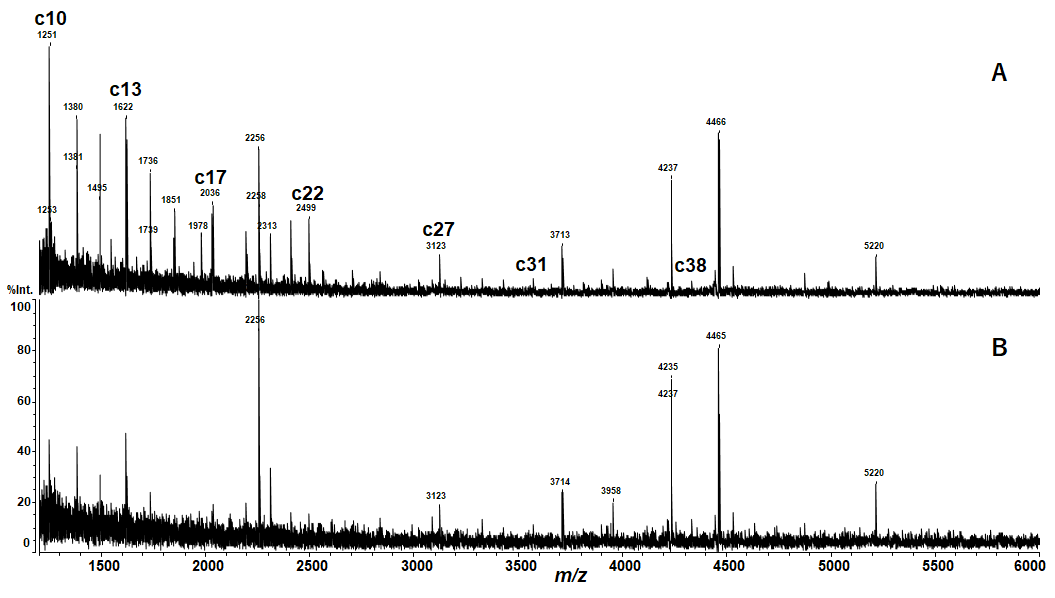


**Fig. S4.** MALDI mass spectra of αLA with DTT/AcOHNH_3_ and DTT/TFA for (A) 30 min and (B) 4 days of incubation.


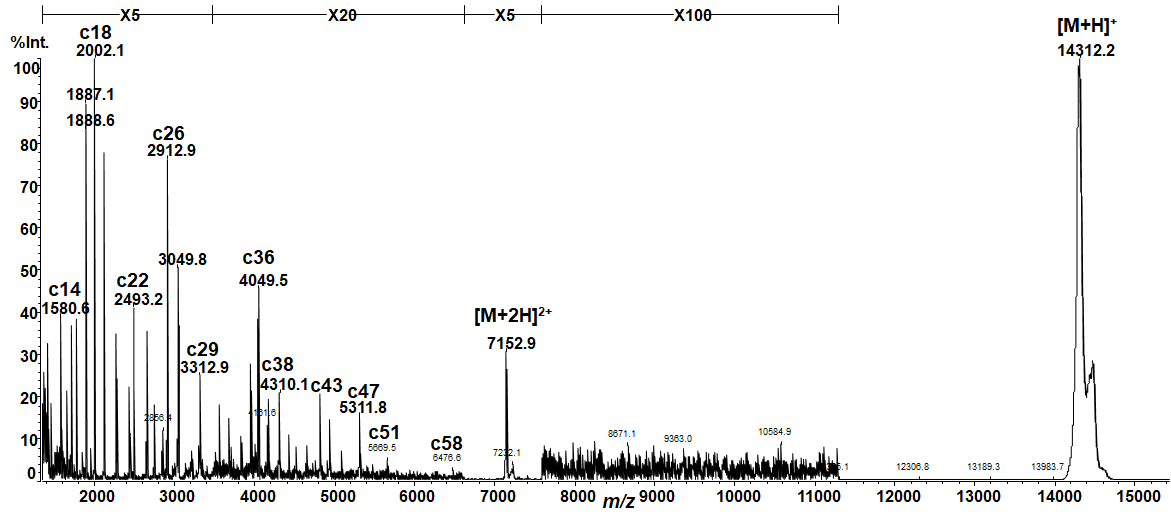


**Fig. S5.** MALDI mass spectrum of HEL with DTT/TFA for 4 hr of incubation.
